# Effect of Hardener Type on the Photochemical and Antifungal Performance of Epoxy and Oligophosphonate S–IPNs

**DOI:** 10.3390/polym14183784

**Published:** 2022-09-09

**Authors:** Cristian-Dragos Varganici, Liliana Rosu, Dan Rosu, Corneliu Hamciuc, Irina Rosca, Ana-Lavinia Vasiliu

**Affiliations:** “Petru Poni” Institute of Macromolecular Chemistry, 41A Grigore Ghica Voda Alley, 700487 Iasi, Romania

**Keywords:** epoxy resin, oligophosphonate, semi-interpenetrating polymer networks, photochemistry, fire performance, antifungal behavior

## Abstract

Due to their highly reactive character and multiple crosslinking capacity, epoxy resins are one of the worldwide market-dominating classes of thermosetting polymers and are present in a wide range of technical applications, including structural adhesives, coatings and polymer matrices for composite materials. Despite their excellent features, epoxy resins are known to be highly flammable and possess low thermal stability and a brittle character and crack easily under impact forces. An efficient approach towards eliminating such drawbacks resides in obtaining epoxy-based semi-interpenetrating polymer networks, which possess excellent control over the morphology. The article describes the comparative effect of three hardeners (aromatic, cycloaliphatic and aliphatic) in the presence of an oligophosphonate (–R–O–PO(C_6_H_5_)–O–) (2 wt.% phosphorus) on the photochemical, fire and antifungal performance of bisphenol A diglycidyl ether semi-interpenetrating polymer networks. The networks are designed as future potential outdoor protective coatings for different substrates. The fire resistance capacity of the networks was undertaken with microscale combustion calorimetry before and after photochemical aging. Structural changes during photoirradiation were monitored via color modification studies, Fourier-transform infrared spectroscopy, differential scanning calorimetry, morphological assessment through scanning electron microscopy and mass loss measurements in order to propose the action mode of the hardeners and the oligophosphonate on the material properties. Microbiological testing was also undertaken with the aid of three specific wood decaying fungi as a first substrate.

## 1. Introduction

Emerging high-tech fields continuously require the development of high performance materials with a gainful ratio between end-use capabilities and production costs. This aspect reorients research facilities and production industries towards (bio-based) multicomponent polymer materials [1,2]. Semi-interpenetrating polymer networks (S–IPNs) are a class of particular blends. Their unique structures consist of two or more intertwined chemically dissimilar components of which at least one is crosslinked and the other linear, with no chemical bonding between them [3,4]. 

Among other classes of related multicomponent polymer materials (e.g., blends, grafts and blocks) S–IPNs possess the capacity of curing, allowing exceptional morphological control. The obtaining of S–IPNs permits the occurrence of a synergic effect that generates a forced phase compatibilization of the components, leading to the improvement in the properties of the initial components [5,6]. 

The highly reactive and versatile oxirane ring possesses the capacity of crosslinking with a wide palette of different compounds for multiple applications [7,8,9,10,11], making epoxies one of the most sought and applied classes of thermosetting polymers [12,13]. Unfortunately, these aspects are not without their drawbacks. 

Epoxy resins are mostly brittle, easy crack under mechanical stress, are low thermally stable and extremely flammable [14]. Although mixing epoxies with different additives or blends may resolve such drawbacks while simultaneously avoiding complicated synthesis and often toxic pathways, the mixing process usually leads to low component miscibility. Hence, the use of S–IPNs may surpass such impediments [4]. Concerning the fire and thermal resistance improvement of epoxy resins, of all described modification strategies [15,16,17,18,19] the adding of organophosphorus additives demonstrated the highest efficiency also in reducing the number of toxic volatiles evolved during combustion [20]. 

In order to improve the mechanical properties of epoxy resins, several modification strategies have been implied, including the addition of nanofillers, rubbers and thermoplastics. Although these added compounds enhanced the toughness, they reduced the thermal stability and elastic modulus and increased the viscosity of the epoxy matrices [21,22,23]. Such serious issues further require the developing of IPNs/S–IPNs for their previously mentioned advantages. 

Phosphorus monomers and additives containing the polar P=O entity are known to enhance the flame retardancy and adhesion of different materials to various substrates. Of these groups, 9,10-dihydro-9-oxa-10-phosphaphenanthrene-10-oxide (DOPO) is vastly applied for different applications [24,25,26,27,28]. The chemical structures, synthesis methods and applications of phosphonates were detailed in a review by Chen and Wang [29].

The article describes for the very first time a study on the photochemical and antifungal behavior of epoxy and oligophosphonate S–IPNs cured with three different hardeners (aromatic, cycloaliphatic and aliphatic). The comparative influence of the three hardeners on the color modifications during irradiation were correlated with structural changes, assessed by Fourier transfer infrared spectroscopy (FT-IR) and differential scanning calorimetry (DSC). Morphological studies before and after photochemical and fungal exposures were conducted by scanning electron microscopy (SEM). 

Being designed in a first instance as potential future wood protective coatings, the S–IPNs were tested and found to be resistant to three specific wood decaying strains: *Cladosporium cladosporioides* (*C. cladosporioides*)*, Penicillium chrysogenum* (*P. chrysogenum*) and *Aspergillus brasiliensis* (*A. brasiliensis*). The influence of UV irradiation on the flame resistance capacity of the S–IPNs was conducted with the aid of microscale combustion calorimetry (MCC). It was observed that, although highly flame retardant, the S–IPNs possessed not great photochemical stability. Thus, the final scope of the research will consist in finding an adequate photostabilizing method and compound for the described samples.

## 2. Experimental Section

### 2.1. Materials

4-Aminophenol, terephthalaldehyde, 4-toluenesulfonic acid (catalyst) and ethanol (solvent), for the use in the first intermediate pathway to the synthesis of the oligophosphonate (OP), were products of Sigma-Aldrich (Darmstadt, Germany) and used as received. DOPO, used in the second intermediate pathway to synthesis of the OP (i.e., the synthesis of the precursor to the OP), was a product of Chemos GmbH (Altdorf, Germany) and it was dehydrated at 150 °C for 3 h before use. 

The curing agents for the epoxy resin: 4,4′-diaminodiphenylsulfone (DDS), 1,3-bis(aminomethyl)cyclohexane (CYDM), octamethylenediamine (8CH_2_DA) and the phenylphosphonic dichloride, applied in the synthesis of the oligophosphonate (OP), were products of Sigma-Aldrich (Darmstadt, Germany) and were used as received. N-methyl-2-pyrrolidone (solvent) and triethylamine (acid acceptor), necessary for the synthesis of the OP, were products of Fluka (Munich, Germany) and used as received. Diglycidyl ether of bisphenol A epoxy resin (EP) from Sigma-Aldrich (St. Louis, MO, USA) was used, having an epoxy equivalent weight 0.53 equiv. per 100 g (M_n_~377 g mol^−1^; viscosity of 15 Pa⋅s; density of 1160 kg m^−3^ at 25 °C). 

The synthesis and structural characterization of the OP was detailed in a previous work [30]. The synthesis procedure is overviewed in the Supporting Information (see Sections S1.1 and S1.2, Scheme S1).

### 2.2. Obtaining of the S–IPNs

**Scheme 1:**
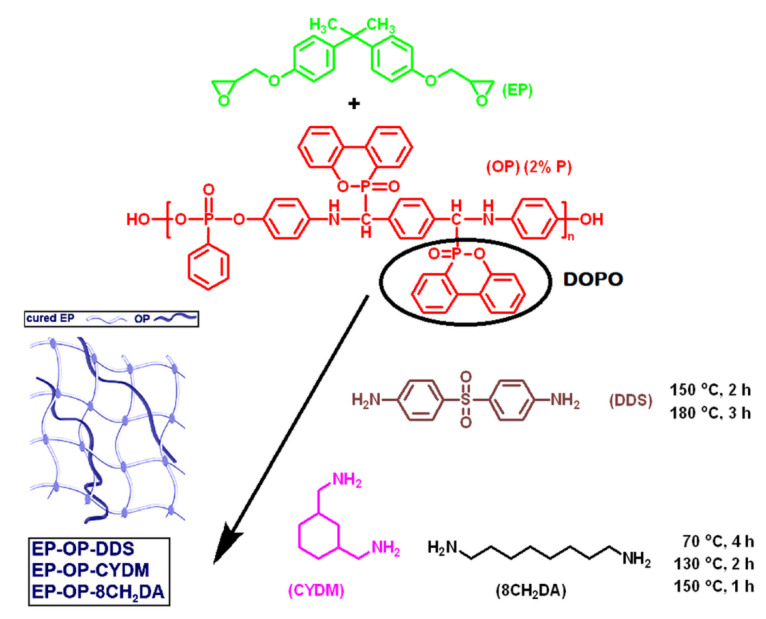
Obtaining of the S–IPNs.

The obtaining and structural characterization of the S–IPNs was described in a recent paper [30]. The procedure [28] implied the mixing of EP with calculated amounts of OP to obtain final products with 2 wt.% phosphorus (Table 1, Scheme 1). The mixing was undertaken under stirring and heating at 130 °C to reach a molecular level of mixing, followed by curing in an oven in the presence of a hardener (DDS, CYDM and 8CH_2_DA) (2:1 epoxy:amine ratio) and cooling the reaction mass to 80 °C (Scheme 1). The homogeneously stirred formulations were poured into Teflon coated plate shapes of 13 × 125 × 3 mm^3^. The curing protocol for the mixture with DDS was 150 °C for 2 h and 180 °C for 3 h and for the other two formulations was 70 °C for 4 h, 130 °C for 2 h and 150 °C for 1 h, followed by slow cooling to room temperature in order to prevent cracking.

### 2.3. Measurements

#### 2.3.1. Structural Characterization of the OP

The synthesis of the OP was undertaken via solution policondensation between equimolecular quantities of the monomers bis((6-oxido-6H-dibenz [c,e][1,2]oxaphosphorinyl)-(4-hydroxyaniline)-methylene)-1,4-phenylene and phenylphosphonic dichloride in N-methyl-2-pyrrolidone as solvent. The syntheses of the intermediates and the OP are detailed in the Supplementary Materials (Section S1).

The structural characterization of the OP was undertaken by means of proton and phosphorus 31-nuclear magnetic resonance (^1^H-NMR, ^31^P-NMR), inherent viscosity measurements and gel permeation chromatography (GPC) (M_n_ = 6184 g mol^−1^; M_w_ = 6501 g mol^−1^; M_w_/M_n_ = 1.051). For the OP powder, the Fourier-transform infrared spectroscopy (FT-IR) spectrum was registered on a Brüker Vertex 70 device (Karlsruhe, Germany) on KBr pellets. All the structural characterizations of OP were reported in a previous article [30].

#### 2.3.2. Irradiation

The samples were exposed up to 500 h of UV irradiation with the aid of a UVP-B-100AP high pressure mercury lamp (100 W), provided with an optical filter with maximum transparency at 365 nm (Analytik Jena, Jena, Germany), equipped with a fan. The irradiance values at 365 nm were measured with a PMA 2100 radiometer (Solar Light Company, Glenside, PA, USA) equipped with a PMA 2110 detector with spectral response between 320 and 400 nm (UVA), and the value at the sample surface was 110 W m^−2^. 

The radiant exposure measured for 1 h of irradiation was 161.5 kJ m^−2^. These values approximate 305 days of exposure to natural light simulated into 500 h of laboratory accelerated UV exposure. This is the mean radiant exposure value typical for 3 months in Iasi, Romania (47.18° N, 27.55° E). The irradiation was performed in air, at 25 ± 3 °C and a relative humidity of 40%. The samples were extracted from the irradiation device at intervals of 100, 200, 300, 400 and 500 h and analyzed.

#### 2.3.3. Color Modification Studies

**Scheme 2:**
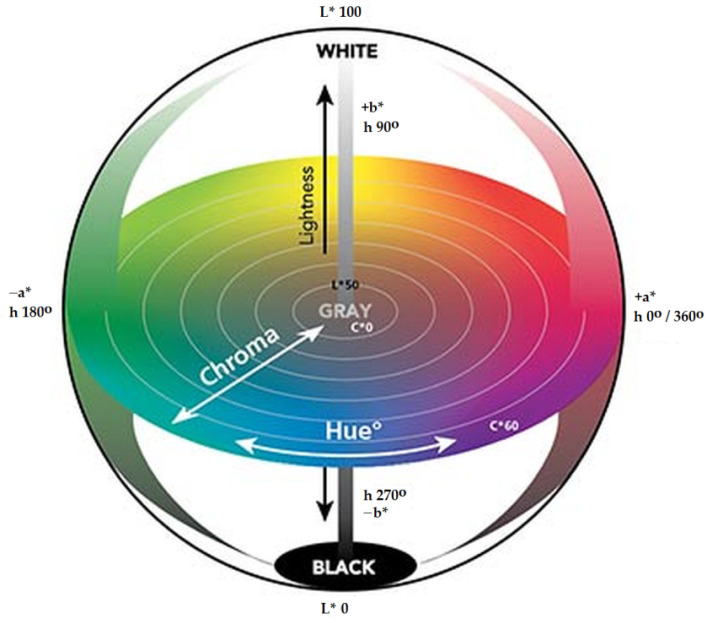
The C.I.E.L*C*b* system is denoted as a sphere containing hundreds of thousands of different colors, each with its unique point within the sphere.

The color changes during UV irradiation were monitored according to the C.I.E.L*a*b* and C.I.E.L*C*h* systems (Scheme 2). From Scheme 2, in the C.I.E.L*a*b*, all colors are represented on three perpendicular axes. The vertical axis represents the lightness factor (L*) with values between 0 (representing absolute black) and 100 (representing absolute white). Intermediate values describe different shades of gray. The other two axes are positioned horizontally. 

One axis represents the chromatic parameter of a* with values between −a* and +a*, the negative values describing different shades of green and the positive ones different shades of red. A second axis is denoted b* with which the colors yellow (on the positive direction of the axis) and blue (on the negative direction) were represented. In case of changes in color parameters, the absolute values describe the intensity of the respective colors. At the intersection of the three axes, the samples appear uncolored, and were characterized by different shades of gray [31].

The second system used for color characterization uses polar coordinates that are denoted C.I.E.L*C*h*. Along with the parameters described in the C.I.E.L*a*b* system, the C.I.E.L*C*h* also quantifies the angle at which the colors are observed. The value C* (chroma) is the chromatic and h* describes the hue, an angular size. Between the chromatic parameters used in the two systems there exist the following relations:

(1) $$C^{*}=\sqrt{\left({a*}^{2}+{b*}^{2}\right)}$$

(2) $$h^{*}=arctan\left(\frac{b}{a}\right)$$

A global reflection on the influence of UV exposure on the color parameters is represented by the values Δ*E* calculated with the Equations (3) and (4), in which, by ΔL*, Δa*, Δb* and ΔC* are noted the differences between the chromatic parameters of the irradiated and non-irradiated samples, as measured at certain time intervals.

(3) $${\Delta E}_{L*,a*,b*}=\sqrt{{\Delta L*}^{2}+{\Delta a*}^{2}+{\Delta b*}^{2}}$$

(4) $${\Delta E}_{L*,C*h*\;}=\sqrt{{\Delta L*}^{2}{\Delta C*}^{2}+{\Delta h*}^{2}}$$

#### 2.3.4. Structural Modifications by FT-IR during Photoirradiation

The S–IPN films were analyzed with a MIRacle^TM^ crystal plate for attenuated total reflectance (ATR), manufactured from diamond. The spectra were recorded in the range 4000–600 cm^−1^ at a resolution of 4 cm^−1^ and 64 scans.

#### 2.3.5. Morphological Study

A scanning electron microscope SEM Quanta 200 (Hillsboro, OR, USA) was used to assess the comparative morphology of the S–IPNs before and after UV irradiation and microbiological attack. The device was operating at 30 kV with secondary and backscattering electrons and in high vacuum mode.

#### 2.3.6. Differential Scanning Calorimetry

Differential scanning calorimetry (DSC) measurements were conducted on a 200 F3 Maia device (Netzsch Gerätebau GmbH, Selb, Germany). A total of 9 mg of each sample was weighed and added into aluminium crucibles and sealed shut with pierced lids. Nitrogen was used as inert gas (flow 50 mL min^−1^) at a heating rate of 10 °C min^−1^. The glass transition temperature (T_g_) domains of the samples were extracted.

#### 2.3.7. Microscale Combustion Calorimetry (MCC)

The heat of combustion measurements were recorded on a micro scale combustion calorimeter (MCC) apparatus (Fire Testing Technology Instrument) (West Sussex, UK). Approximately 6 mg of sample were heated in a mixed atmosphere (oxygen 80 mL min^−1^; nitrogen 20 mL min^−1^) from 40 to 600 °C at a heating rate of 1 °C s^−1^. The evolved gaseous volatiles were swept into a combustor and oxidized. The results were reported as average values from three measurements.

#### 2.3.8. Antifungal Behavior

The samples resistance was tested against three different typical wood decaying fungal strains: *C. cladosporioides* ATCC16022, *P. chrysogenum* ATCC10106 and *A. brasiliensis* ATCC 9642. The samples were placed on Petri dishes with proper media represented by malt extract agar (MEA) for *P. chrysogenum* and potato dextrose agar (PDA) for the other two strains, dishes inoculated with a fixed amount of test-microorganisms (0.5 McFarland standards optical turbidity in sterile saline solution, yielding a suspension containing approximately 1 × 10^8^ CFU mL^−1^ for the three microorganisms) and afterwards incubated for 10 weeks at 25 °C.

The visual aspect of the fungi colonies on the samples surfaces was monitored at each 7 days interval, during 10 weeks of incubation. After 10 weeks of exposure to fungal attack, the samples were extracted from the culture plates and the mycelium present on their surfaces was rinsed repeatedly and pipetted into 1.5 mL Eppendorf tubes. 

Then, the S–IPNs were accurately weighed to highlight the retained moisture. The wet samples were oven–dried at 100 °C for 3 h until they reached constant mass. The previously resulted suspension was serially diluted and, in order to quantify the anti-adherence (fungistatic) properties of the samples against fungal attack it was used a CellTiter 96 AQueous One Solution Cell Proliferation Assay, MTS (Promega, Madison, WI, USA), by following the protocol recommended by the manufacturer. The experiments were conducted in 96-well culture plates, incubating the suspensions with fungal control for 72 h at 25 °C.

MTS Assay Kit uses a colorimetric method for the sensitive quantification of viable cells. After 72 h, MTS reagent was added 1–3 h prior to absorbance readings. After the formazan formation, the final reading was performed at 490 nm on a FLUOstar^®^ Omega microplate reader (BMG LABTECH, Ortenberg, Germany). Experiments were done in triplicate and cell viability was expressed as percentage of control cells’ viability. Graphical data were expressed as the means ± standard error of the mean.

## 3. Results and Discussions

### 3.1. Color Modifications during UV Irradiation

#### 3.1.1. Lightness Factor Variation (L*)

**Figure 1:**
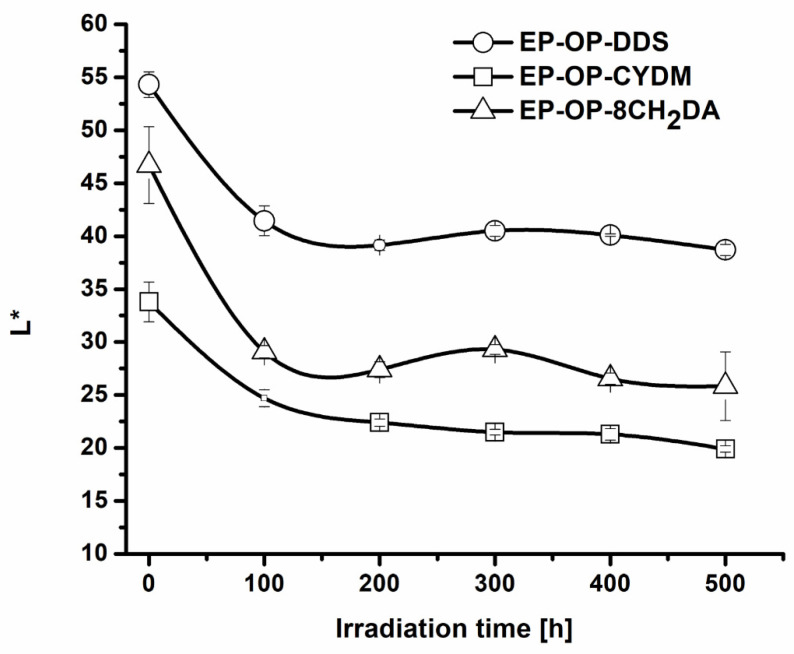
Variation of the L* parameter with irradiation time.

The lightness factor is dependent on the structure of the samples. As it can be seen from Figure 1, the highest values were registered for the EP–OP–DDS S–IPN sample recorded before exposure, for which a mean value of L* = 54.3 was found. The brightness of the sample gradually decreased to 38.7 (ΔL* = −15.6) after 500 h of UV exposure. Therefore, the negative value of ΔL* is an indication of sample darkening during the 500 h of exposure. For the EP–OP–8CH_2_DA sample, an initial average value of L* was found to be lower (L* = 46.7), with the surface of the material darker than EP–OP–DDS.

In this case, the sample is blackened during irradiation, so that after 500 h of exposure the measured value of L* is reduced to 29.1 (ΔL* = −20.9). The more negative value of ΔL* indicates a higher darkening tendency of S–IPN EP–OP–8CH_2_DA compared to EP–OP–DDS. The EP–OP–CYDM S–IPN is characterized by the lowest initial L* value (L* = 33.8). However, the L* value decreased the least after 500 h of exposure, being 19.9 (ΔL* = −13.9).

#### 3.1.2. Variation of Chromatic Parameter a* during UV Irradiation

**Figure 2:**
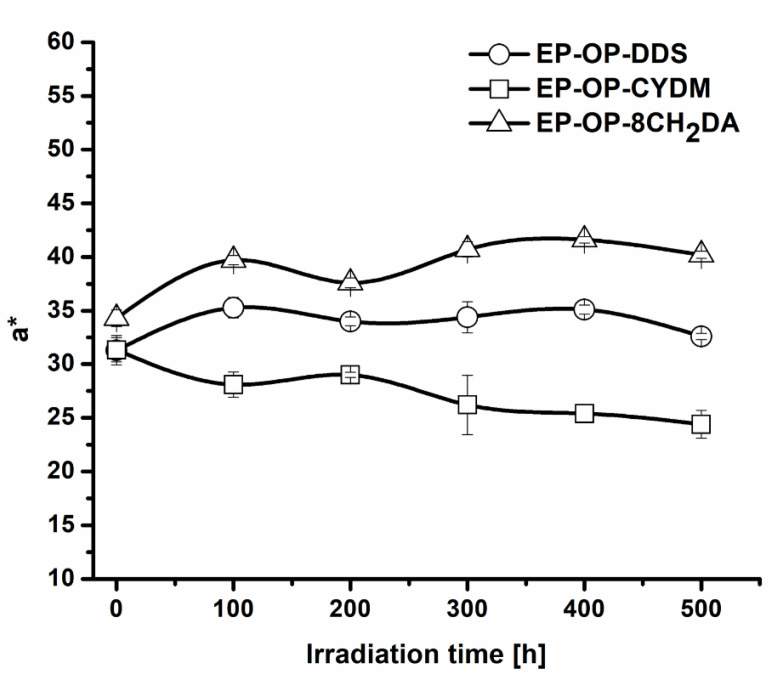
Variation of the a* parameter with irradiation time.

The variation of the chromatic parameter a* with irradiation time is shown in Figure 2. The chromatic parameter describing the red ↔ green axis (a*) increases slightly with exposure time for EP–OP–DDS (Δa* = 4.47) and EP–OP–8CH_2_DA (Δa* = 7.26) samples after 400 h of UV exposure, correlated with accumulation of red chromophores on the surface. However, the new colored structures are photochemically unstable, because prolonging the exposure to 500 h reduces the differences to 1.94 for EP–OP–DDS and to 5.86 for EP–OP–8CH_2_DA. The UV irradiation decreases the red color saturation for S–IPN EP–OP–CYDM. The a* values are negative during the entire exposure time, and the differences are maximal after 500 h (Δa* = −6.92).

#### 3.1.3. Variation of Chromatic Parameter b* during UV Irradiation

**Figure 3:**
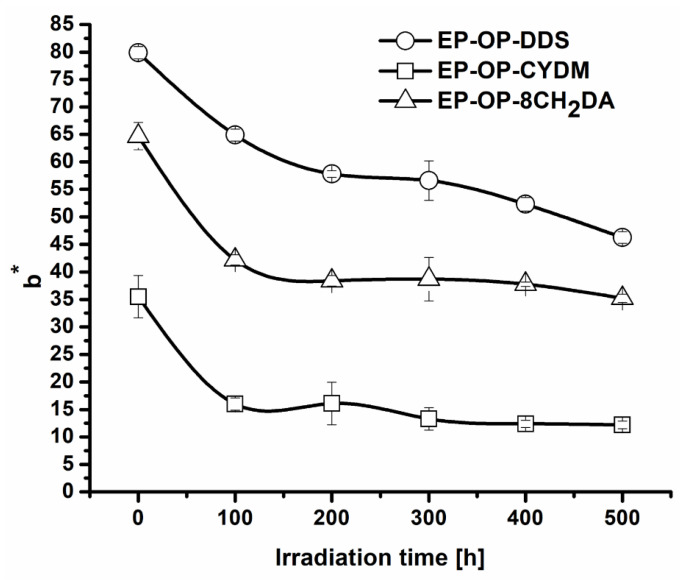
Variation of the b* parameter with irradiation time.

The parameter b*, representing the yellow ↔ blue color axis, shows only positive values (Figure 3). This aspect indicates the formation of yellow chromophores on the surfaces of the S–IPNs, whose concentration depends on their structure. The color saturation of the non-irradiated samples decreases in the order: EP–OP–DDS > EP–OP–8CH_2_DA > EP–OP–CYDM. However, yellow chromophores are unstable to UV action [32]. The b* values decrease continuously and faster in the first 100 h of irradiation, with Δb* values ranging between −15.0 for EP–OP–DDS and −22.6 for EP–OP–8CH_2_DA. The S–IPN EP–OP–CYDM had and intermediate Δb* value (−19.6) at the end of UV exposure. The Δb* values specific to the irradiated samples were: −33.5 for EP–OP–DDS, −29.5 for EP–OP–8CH_2_DA and −23.3 for EP–OP–CYDM.

#### 3.1.4. Variation of Chroma Parameter C* during UV Irradiation

**Figure 4:**
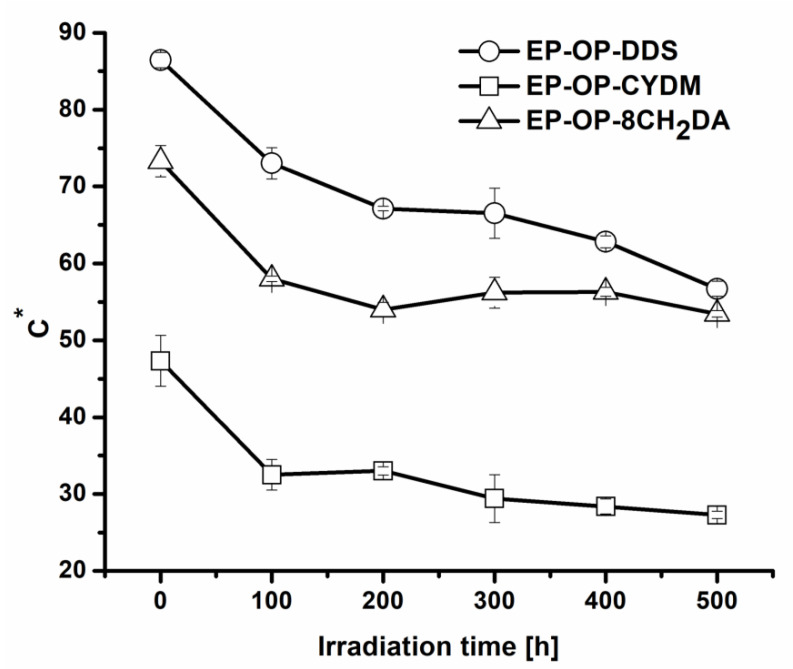
Variation of the chroma parameter with irradiation time.

The simultaneous change of chromatic parameters as a result of UV exposure can be described using the Chroma parameter (C*). Figure 4 shows that UV radiation causes a rapid decrease in C* values in the first 100 h of exposure, after which the degradation of chromatics occurs somewhat slower. The observations showed that all ΔC* values were negative: −19.9 for EP–OP–8CH_2_DA, −20.1 for EP–OP–CYDM and −28.7 for EP–OP–DDS.

#### 3.1.5. Variation of Δ*E* Values during UV Irradiation

A global reflection on the influence of UV exposure on the color parameters is represented by the values ΔE calculated with the relations (3) and (4), in which, by ΔL*, a*, Δb* and ΔC* are the differences between the chromatic parameters of the irradiated and non-irradiated samples measured at certain time intervals. The results are given in the Supporting Information (see Table S1).

**Figure 5:**
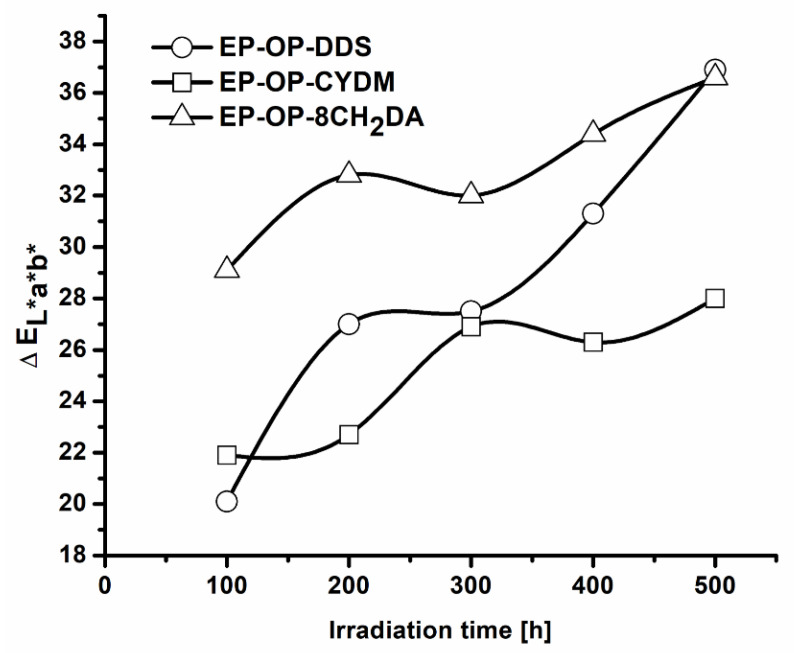
Color differences variation with exposure time.

Table S1 and Figure 5 show that both ΔE_L__*a*b*_ and ΔE_L__*C*h*_ values increase in parallel with UV exposure time. It can be seen that, after 500 h of exposure, the largest global color changes were recorded in the case of the S–IPN EP–OP–8CH_2_DA followed by EP–OP–DDS, while for the EP–OP–CYDM sample the global changes are smaller. One can also notice a good concordance between the ΔE values calculated by the two methods, the values being comparable. Table S2 (see Supporting Information) compares the colors of the studied samples measured before and after 500 h exposure time at 365 nm accompanied by the appropriate color parameters. The mass variation during UV irradiation is discussed in the Supporting Information (see Section S2.1, Figure S1).

### 3.2. Structural Modifications

#### FT-IR Analysis

**Figure 6:**
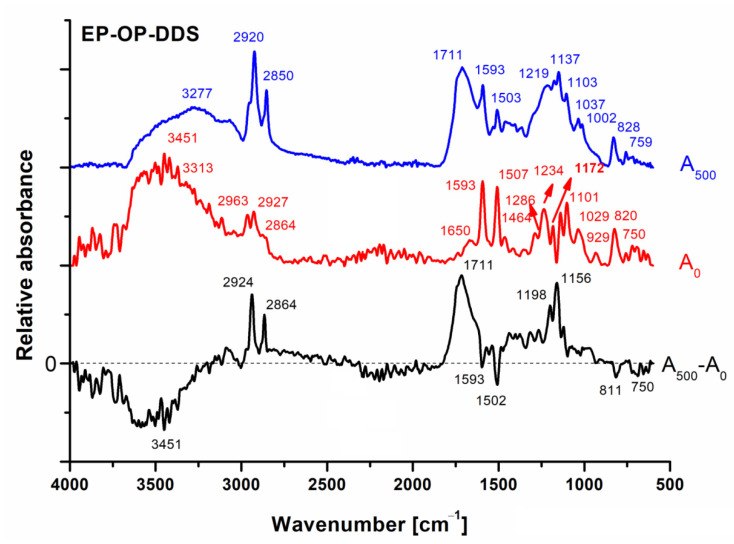
FT-IR spectra of the S–IPN EP–OP–DDS recorded before (A_0_) and after 500 h exposure (A_500_) and the difference spectrum (A_500_–A_0_).

**Figure 7:**
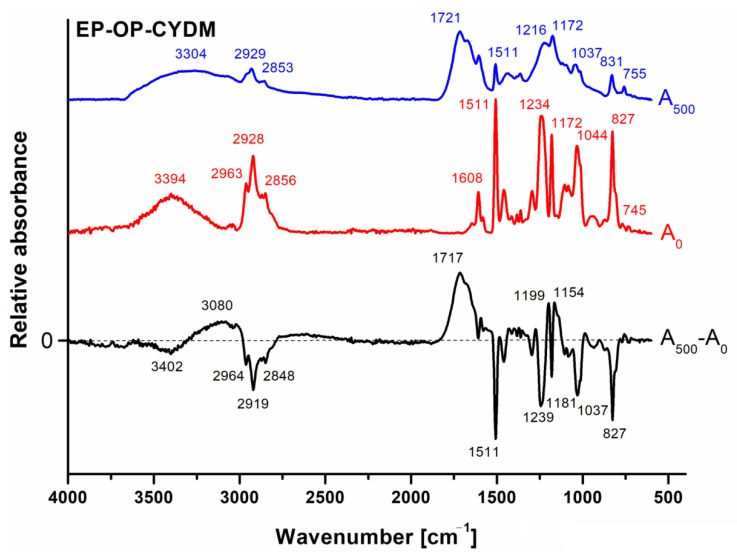
FT-IR spectra of the S–IPN EP–OP–CYDM recorded before (A_0_) and after 500 h exposure (A_500_) and the difference spectrum (A_500_–A_0_).

**Figure 8:**
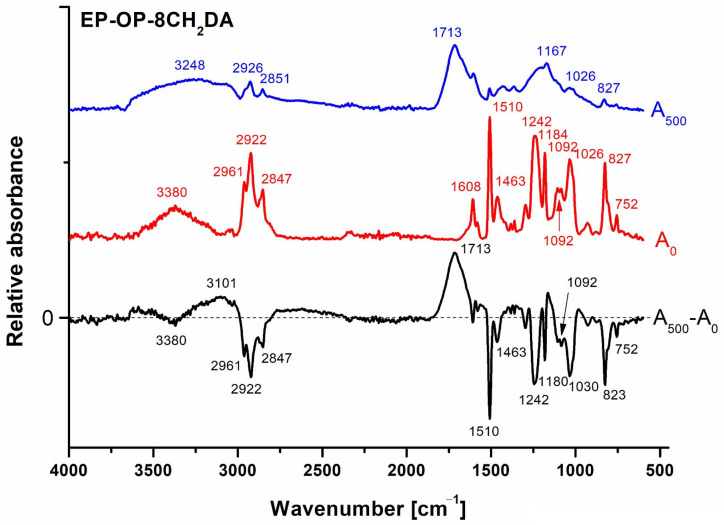
FT-IR spectra of the S–IPN EP–OP–8CH_2_DA recorded before (A_0_) and after 500 h exposure (A_500_) and the difference spectrum (A_500_–A_0_).

Figure 6 shows the FT-IR spectra of the S–IPN EP–OP–DDS recorded before and after UV exposure, and the difference spectrum between the absorbance of irradiated and non-irradiated samples. The difference spectra in Figure 6, Figure 7 and Figure 8 display positive signals (oriented upwards) and negative signals (oriented downwards). The positive signals indicate the entities newly formed during irradiation. Negative signals indicate the degraded structures during irradiation. The decrease in signals shows that photodegradation takes place through mass loss and chain scission [33].

In the FT-IR spectrum of the non-irradiated EP–OP–DDS sample (A_0_), several groups of signals can be identified. These are located both in the area of the functional groups (1500–4000 cm^−1^) and of the fingerprint (600–1500 cm^−1^). The wide signal in the range 3082–3700 cm^−1^ with the peaks at 3451 cm^−1^ and 3313 cm^−1^ can be attributed to the tensile vibrations of the N–H and O–H bonds resulting from the reaction of the crosslinking agent (DDS) with the oxirane ring of the epoxy component. 

The signal group located at 2963 cm^–1^, 2864 cm^–1^ and 2927 cm^−1^ is specific to the valence vibrations of the CH_2_ and CH_3_ groups in the epoxy (EP) component. The signalsfrom 1593 cm^−1^, 1507 cm^−1^ and 750 cm^−1^ can be attributed to the vibrations of the C=C bonds specific to the aromatic cycles from both oligophosphonate and epoxy resin. The signal from 1172 cm^−1^ is specific to the C–N bond that appeared after the crosslinking of the epoxy resin, while the signal from 1464 cm^−1^ is characteristic to the valence vibration of the P–C bond. The valence vibrations of the P–O–C bonds appear in the S–IPN EP–OP–DDS spectrum at 1029 cm^−1^ and 929 cm^−1^. The P=O bond in DOPO is represented by the signal at 1234 cm^−1^.

The FT-IR spectrum of the EP–OP–DDS recorded after 500 h irradiation (A_500_) is also given in Figure 6. The analysis of both the spectrum recorded after UV exposure and the difference spectrum (A_500_–A_0_) shows a decrease in signal intensity with the peak at 3452 cm^−1^ accompanied by the flattening and shift of the peak towards a smaller wavenumber value (3277 cm^−1^). This aspect indicates the presence of new O–H groups generated during irradiation and their involvement in hydrogen bonds formation. The epoxy resin and/or hardener possess groups sensitive to photo-oxidation (–CH<, –CH_2_–, etc.). The photo-oxidative degradation of the epoxy resin and/or hardener via hydroperoxide intermediates, with the formation of O–H groups, was thoroughly discussed in the literature [34,35]. 

The intensification of the signals are characteristic to the CH_2_ and CH_3_ moieties, which may result from the scission of the epoxy skeleton with the formation of smaller fragments [36,37]. A strong photooxidative effect of UV radiation on the EP–OP–DDS sample is highlighted by the presence of the broad signal from 1821–1630 cm^−1^ with the peak located at 1711 cm^−1^, indicating a complex mixture of photodegradation products with carbonyl structure (anhydrides, aldehydes, ketones, carboxylic acids) [38]. The weak negative signals with the peaks at 1593 cm^−1^, 1502 cm^−1^ and 750 cm^−1^ represent some minor changes of the C=C bonds from aromatic nuclei.

Figure 7 shows the FT-IR spectra of the S–IPN EP–OP–CYDM recorded before and after UV exposure, and the difference spectrum between the absorbance of irradiated and non-irradiated samples.

In Figure 7, there occur significant changes in the FT-IR difference spectrum after irradiation in the sense of decreasing signal strength, which means loss of chemical structures, such as of amino groups in the structure of the curing agent, marked by the negative signal from 3402 cm^−1^. It can also be seen a decrease in the intensity of the signals in the area 2964–2848 cm^−1^ specific to the valence vibrations of the alkyl groups, also indicating main chain scission. Another observation is the significant decrease in signal strength from 1511 cm^−1^, which corresponds to the tensile vibrations of the C=C bonds specific to the aromatic nuclei. 

It is as if the S–IPN structure fragments during irradiation with the formation of low molecular weight compounds that subsequently detach from the main structure. Such a phenomenon can be correlated with the significant mass loss recorded during irradiation. Another observation is the significant widening of signals after irradiation, which can also be explained by the fragmentation of the main chain of S–IPNs and the formation of new chemical compounds with smaller dimensions. 

As in the case of the previously analyzed product (EP–OP–DDS), after irradiation, there was found a new signal with a wide peak at 1717 cm^−1^, which can be identified both in the spectrum of the irradiated sample (1721 cm^−1^) and in the difference spectrum (1717 cm^−1^). The signal can be attributed to the appearance of new structures with carbonyl groups. The observation of intense photooxidation phenomena is thus supported.

Figure 8 shows the FT-IR spectra of the S–IPN EP–OP–8CH_2_DA recorded before and after UV exposure, and the difference spectrum between the absorbance of irradiated and non-irradiated samples. By analyzing the difference spectrum in Figure 8, one may also identify the presence of signals in the positive zone that characterize the new chemical structures formed as a result of UV exposure, as well as negative signals that describe the decomposed chemical structures. 

The wide positive signal from 3101 cm^−1^ can be attributed to new hydroxyl groups and the wide one from 1713 cm^−1^ supports the appearance of carbonyl functions with various structures (i.e., organic acids, aldehydes, ketones and peroxides) as a result of photooxidation. Negative signals indicate the breakdown of the polymer in the sense that some of the chemical structures that vibrate at 3380 cm^−1^ (N–H for example) are consumed during irradiation, as is the case with the vibrations of C–H groups in the CH_2_ and CH_3_ entities in the range 2296–2847 cm^−1^.

It is possible that these fragments also come from the structure of the 8CH_2_DA curing agent. Significant vibration reduction from 1180 and 1172 cm^−1^ characterizing the C–N bond in the crosslinked epoxy resin can also be observed. The signal from 1463 cm^−1^, characteristic to the C–P bond, decreased after UV exposure, signifying the loosening of this bond. The difference spectrum indicates the almost complete disappearance of the signals from 1510 cm^−1^ and 750 cm^−1^, which are specific to the double C=C bonds of the aromatic cycles in polyphosphonate. The negative signals from 1028 and 931 cm^−1^ indicate photodegradation of the P–O–C bond in the S–IPN EP–OP–8CH_2_DA. It can be concluded that the photodegradation the S–IPN EP–OP–8CH_2_DA structure is the result of two processes involving: (1) photooxidation by hydroperoxide intermediates and (2) the photochemical destruction of the C–NH–C and P–O–C crosslinking points.

### 3.3. Morphological and Thermal Characterization

**Figure 9:**
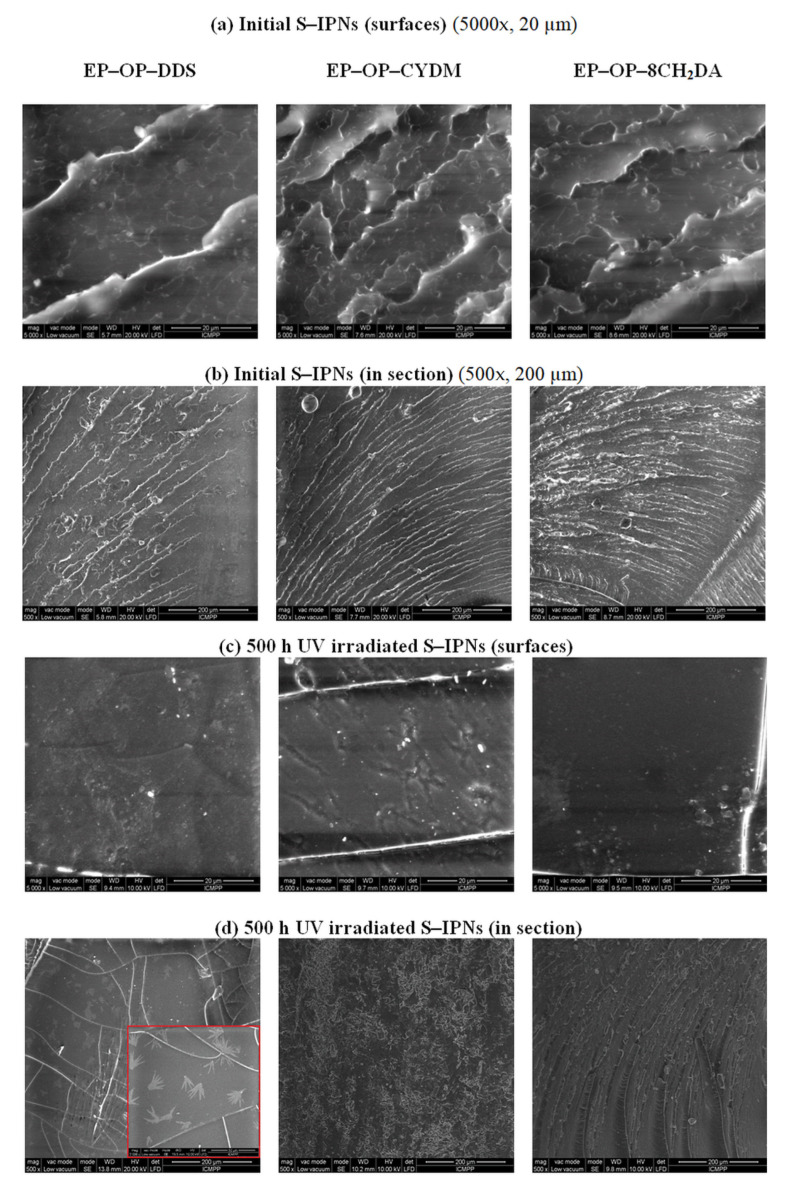
SEM micrographs of the S–IPNs before and after irradiation.

**Figure 10:**
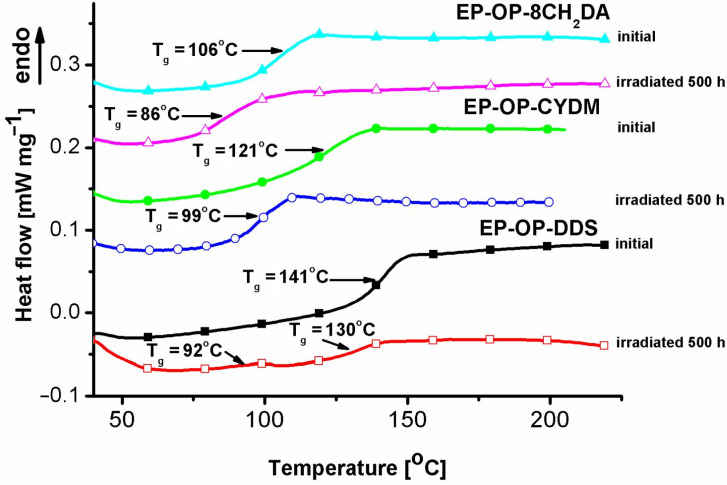
DSC curves of the S–IPNs before and after 500 h of UV irradiation.

Figure 9 and Figure 10 show the SEM micrographs (surface and in section) and DSC curves of the S–IPNs before and after 500 h of UV irradiation, respectively. During the thermal treatment, the OP agglomerates generated in the S–IPNs uniform and parallel fracture lines [30]. (Figure 9a,b). The uniform distribution of the micro fractures in the S–IPNs is an indication of the homogeneous dispersion of the OP into the cured epoxy resin [28]. This is confirmed by the presence of a single glass transition temperature (T_g_) value on the DSC curves (Figure 10).

The aggressive action of the UV irradiation may lead to the generating of visible cracks on the surfaces of the samples and a series of irregular shapes, which seem to appear on both the surface and into the mass of the materials, underneath the physically observable cracks (Figure 9c,d). The decreases of the material masses (see Section S2.1 in the Supporting Information), accompanied by a significant decline in the T_g_ values (11 °C for EP–OP–DDS, 22 °C for EP–OP–CYDM and 20 °C for EP–OP–8CH_2_DA) (Figure 10) after irradiation confirm this aspect. Moreover, the section SEM micrographs of the S–IPNs also indicate discontinuities, and cracks into their linear and relatively uniform aspect (Figure 9d).

The EP–OP–DDS S–IPN exhibits a ‘sea–island’ morphology type of a hand–like shape after UV irradiation (Figure 9d), while the DSC curve of this S–IPN indicates the presence of two T_g_ values, indicating the occurrence of a phase separation phenomenon. The T_g_ value of 92 °C is most likely exhibited by the OP in the EP–OP–DDS S–IPN. The pristine OP was presented in the form of a yellow powder with a T_g_ value at 132 °C [30]. Once introduced into the DDS cured S–IPN and after photoirradiation, the T_g_ of the OP decreases significantly by 40 °C (T_g_ = 92 °C). Nonetheless, the T_g_ of S–IPN EP–OP–DDS decreases by 11 °C compared to the significant comparable decreases in EP–OP–8CH_2_DA (20 °C) and EP–OP–CYDM (22 °C) during irradiation. The thermogravimetric analysis (TGA) is discussed in the Supporting Information (see Section S2.2 and Table S3). It is worth mentioning that the DSC curves show no exothermal profile. This signifies high or even complete crosslinking between the amine groups of the hardeners and the epoxide rings. This aspect is in good agreement with the FT-IR results.

Absolute heat capacities where obtained from the T_g_s. Crosslinking densities of the initial samples were calculated with Equation (5) [39], where $C_{p\;}^{i}$ and $C_{p}^{0}$ are the heat capacities of the S–IPN and that of the linear polymer (OP). The values are given in Table 2.

(5) $${\rho^{\prime }}_{c}=\frac{C_{p\;}^{i}-C_{p}^{0}}{C_{p}^{0}}=\frac{\Delta C_{p}^{i}}{C_{p}^{0}}$$

### 3.4. Flame Resistant Capacity of the UV Irradiated S–IPNs

**Figure 11:**
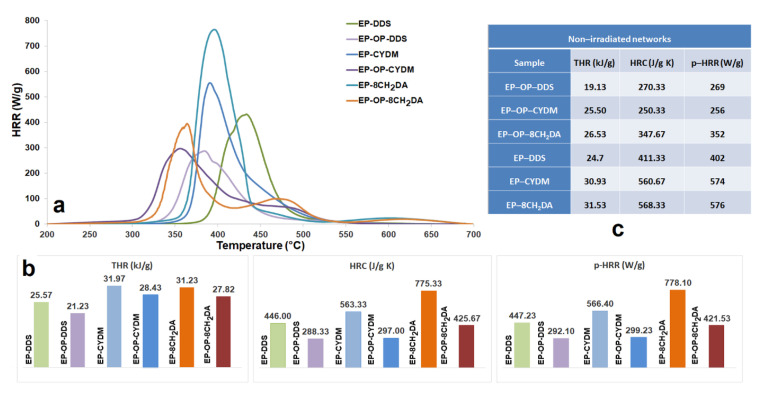
(**a**) HRR and (**b**) MCC data of UV irradiated samples; (**c**) table of MCC data of non-irradiated samples. (**c**) is partially reproduced under Creative Commons license CC BY 4.0 [30].

The flame resistance capacity of the materials was undertaken with the aid of MCC method. MCC is an indispensable technique in studying polymer combustion, providing valuable data regarding fire risks, via parameters such as: the heat release rate (HRR), peak of heat release rate (*p*–HRR) and total heat release (THR). Through the integration of the HRR over the entire time range (conversion into temperature in Figure 11) the total heat capacity (THC) values are yielded. The results are summarized in Figure 11.

The flame retardant character of the non-irradiated S–IPNs was reported in a previous paper and demonstrated through their lower THR and significantly lower heat release capacity (HRC) and *p*–HRR values by comparison with the OP free cured matrices, following both condensed and gas phase mechanisms [30]. In practice, the reduction in the *p*–HRR values increases the time to escape a fire incident. The table from Figure 11c depicts the respective parameters for sake of comparison. From Figure 11c it was reported that the p–HRR values decrease with 55.4% (EP–OP–CYDM vs. EP–CYDM), 38.9% (EP–OP–8CH_2_DA vs. EP–8CH_2_DA) and 33.08% (EP–OP–DDS vs. EP–DDS) and the EP–OP–DDS was the sole network to achieve a UL 94–V0 [30]. As one may observe from Figure 11a–c, UV irradiation leads to a decline in the fire performance of the S–IPNs. This is reflected through the seemingly increased MCC parameters of the irradiated S–IPNs compared to the ones of the initial samples, especially the HRC and *p*–HRR values. The HRC values of the S–IPNs increase: from 270.33 J/g K to 288.33 J/g K for EP–OP–DDS, from 250.33 J/g K to 297 J/g K for EP–OP–CYDM and from 347.67 J/g K to 425.67 J/g K for EP–OP–8CH_2_DA. The *p*–HRR values of the S–IPNs also increase: from 269 W/g K to 292.1 W/g for EP–OP–DDS, from 256 W/g to 299.23 W/g for EP–OP–CYDM and from 352 W/g to 421.53 W/g for EP–OP–8CH_2_DA. The data indicate that the exposure to UV light has a damaging effect on the S–IPNs through complex chain scissions, aspect confirmed by the registered mass losses and significant lowering of the T_g_ in DSC.

### 3.5. The Microbiological Testing of the S–IPNs

**Figure 12:**
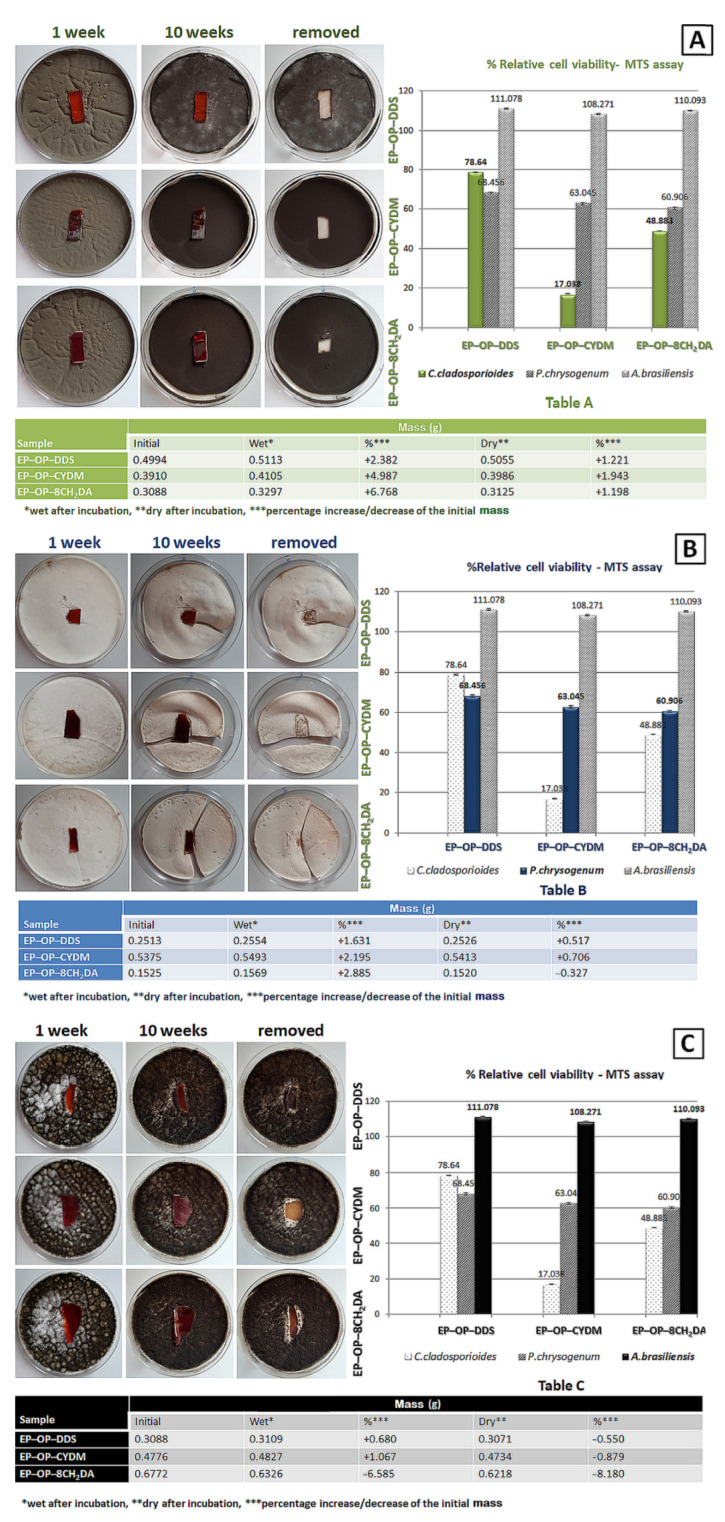
(**A**) S–IPNs exposed to the action of *C. cladosporioides* for 1 week, 10 weeks and removed afterwards; fungistatic efficacy and mass variation. (**B**) S–IPNs exposed to the action of *P. chrysogenum* for 1 week, 10 weeks and removed afterwards; fungistatic efficacy and mass variation. (**C**) S–IPNs exposed to the action of *A. brasiliensis* for 1 week, 10 weeks and removed afterwards; fungistatic efficacy and mass variation.

Since the S–IPNs are intended as future wood protective coatings in a first instance, the samples’ microbiological resistance was tested against three wood decaying fungal strains: *C. cladosporioides* ATCC16022, *P. chrysogenum* ATCC10106 and *A. brasiliensis* ATCC9642. The assessment of degradation degree after 10 weeks of exposure was based on visual observations and the measure of the mass loss. In Figure 12A–C, during the decay test, all surfaces of samples were not covered by the fungal colonies, excepting samples exposed to *A. brasiliensis*, where the edges where slightly covered. There was no notable mass loss for any of the tested samples, excepting those incubated with *A. brasiliensis*. Even in this case, the mass loss was not much reduced, around 1% for most of the samples (Table A–C in Figure 12A–C), with the sole exception of EP–OP–8CH_2_DA, which registered 8% mass loss (Table C in Figure 12C).

**Figure 13:**
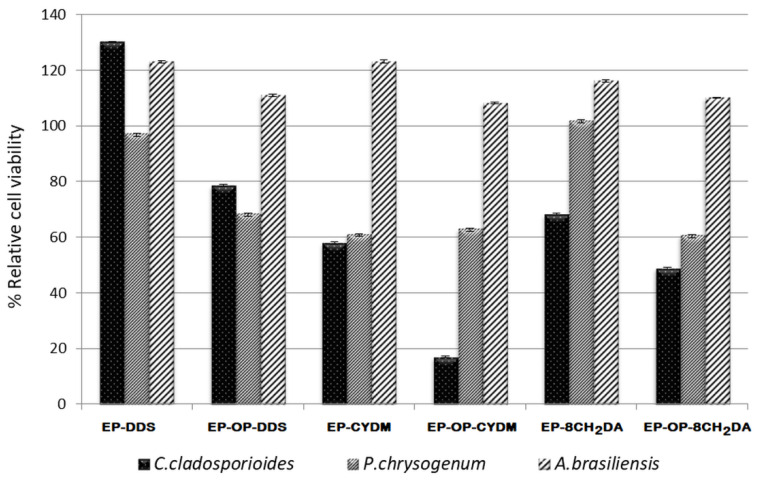
Fungistatic efficacy of the S–IPNs and control samples against the reference strains.

Even if there were not noticed significant mass variations, and also there was not distinguished a notable growth of the colonies on the samples, the fungistatic properties when compared with the control samples (i.e., the crosslinked counterparts without the OP: EP–DDS, EP–CYDM, EP–8CH_2_DA) varied (Figure 13). The S–IPN EP–OP–CYDM reduced the population of *C. cladosporioides* at 17%. Not so promising results were obtained in case of the S–IPNs incubated with *A. brasiliensis*, where there was not noticed a fungistatic activity for all the tested samples.

The samples presented instead fungicidal properties for the samples incubated with *C. cladosporioides* and *A. brasiliensis*, and partially for *P. chrysogenum*, the areas under the removed samples remaining clear of fungal colonies after 10 weeks of incubation, hence the structures adhered perfectly to the culture media (Figure 12A–C).

**Figure 14:**
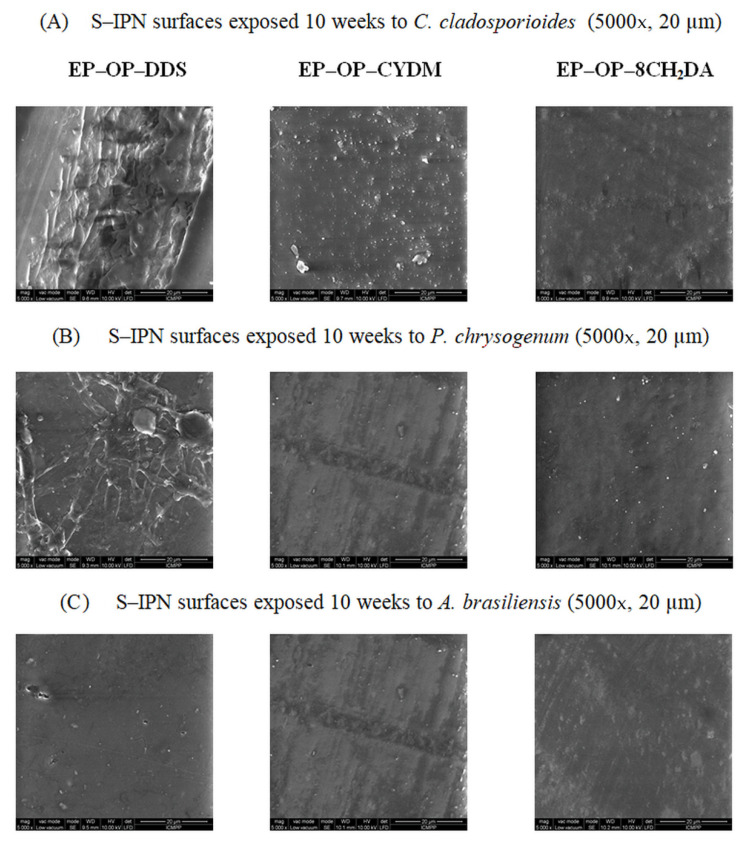
SEM micrographs of the S–IPNs surfaces after fungal attack.

Surface SEM observations recorded after fungal attack confirm that in general the S–IPNs do not show presence of microorganisms (Figure 14), only sporadic mycelial fragments of the tested strains. After the fungal attack the lowest color differences (ΔE*) were registered for samples EP–OP–CYDM and EP–OP–8CH_2_DA incubated with *A. Brasiliensis* (13.2 and 29.5), with the exception of sample EP–OP–DDS where in this case ΔE* was the highest (73.6) (see Table S4 in Supporting Information).

## 4. Conclusions

Flame retardant S–IPNs from an aromatic oligophosphonate and epoxy resin crosslinked with three different curing agents: 4,4′-diaminodiphenylsulfone, 1,3-bis(aminomethyl)cyclohexane and octamethylenediamine were subjected to photo aging. Structural changes during photoirradiation were assessed by color modification studies, FT-IR, DSC, SEM and mass loss measurements. The flame retardant capacity of the structures was assessed with MCC method. The networks exhibited color modifications and structural degradation during UV exposure. This aspect was reflected in morphological modifications, significant decline in T_g_ values and mass losses, especially for the EP–OP–CYDM and EP–OP–8CH_2_DA samples, most probably due to the photochemical instability of the oligophosphonate and aliphatic nature of the hardener, as shown by FT-IR. Microbiological testing showed promising results against the specific wood decaying fungal strains of *C. cladosporioides*, *P. chrysogenum* and *A. brasiliensis*. An overall aspect is that, although a surface phenomenon, UV irradiation contributes to decreasing the flame retardant capacity of the S–IPNs, hence affecting the bulk of the materials. Despite this aspect, the obtained data shows that the S–IPNs are flame retardant. In the present phase of research, the S–IPNs are not yet recommended for outdoor light exposure. It is therefore that the conducted research to this point constitutes a valuable basis towards finding suitable photostabilizers for the S–IPNs.

## Figures and Tables

**Table 1:** Composition of the S–IPNs.

S–IPN	Hardener (g)		OP (g)	EP (g)
EP–OP–DDS	DDS	5.99	5.61	18.36
EP–OP–CYDM	CYDM	3.79	5.56	20.63
EP–OP–8CH_2_DA	8CH_2_DA	3.87	5.65	20.50

**Table 2:** Absolute heat capacities and crosslinking densities.

Sample	*ΔC_p_* (J g^−1^ °C)	*ρ_c_’* (mol cm^−3^)
OP	0.314	–
EP–OP–DDS	0.352	0.121
EP–OP–CYDM	0.335	0.066
EP–OP–8CH_2_DA	0.321	0.023

## Data Availability

Not applicable.

## Supplementary Materials

The following supporting information can be downloaded at: https://www.mdpi.com/article/10.3390/polym14183784/s1, Scheme S1: Synthesis of the intermediates to the OP and the OP; Table S1: The Differences Δ*E_L_*_**a***b**_ and Δ*E_L_*_**C***h**_ Calculated at Different Exposure Times for the Studied S–IPNs; Table S2: Color Modifications During UV Irradiation; Figure S1: Variation of mass loss with irradiation time; Table S3: Thermogravimetric analysis (TGA) Parameters of the S–IPNs; Table S4. Color Parameters Values Modification after Fungal Attack.

## References

[1] Gurunathan T., Nayak S.K. (2016). The Influence of Reactive Organoclay on a Biorenewable Castor Oil-Based Polyurethane Prepolymers Toughened Polylactide Nanocomposites. Polym. Adv. Technol..

[2] Gurunathan T., Chung J.S., Nayak S.K. (2016). Reactive Compatibilization of Biobased Polyurethane Prepolymer Toughening Polylactide Prepared by Melt Blending. J. Polym. Environ..

[3] Cascaval C.N., Ciobanu C., Rosu D., Rosu L. (2002). Polyurethane-Epoxy Maleate of Bisphenol A Semi-Interpenetrating Polymer Networks. J. Appl. Polym. Sci..

[4] Klempner D., Sperling L.H., Utracki L.A. (1994). Interpenetrating Polymer Netwoks.

[5] Thomas R., Vijayan P., Thomas S. (2011). Recycling of Thermosetting Polymers. Recent Developments in Polymer Recycling.

[6] Varganici C.D., Rosu L., Rosu D., Simionescu B.C. (2013). Miscibility Studies of Some Semi–Interpenetrating Polymer Networks Based on an Aromatic Polyurethane and Epoxy Resin. Compos. Part B Eng..

[7] Jian R., Wang P., Duan W., Wang J., Zheng X., Weng J. (2016). Synthesis of a Novel P/N/S-Containing Flame Retardant and Its Application in Epoxy Resin: Thermal Property, Flame Retardance, and Pyrolysis Behavior. Ind. Eng. Chem. Res..

[8] Irfan M.H. (2012). Chemistry and Technology of Thermosetting Polymers in Construction Applications.

[9] Hayaty M., Honarkar H., Beheshty M.H. (2013). Curing Behavior Of Dicyandiamide/Epoxy Resin System Using Different Accelerators. Iran. Polym. J..

[10] Jin F.L., Park S.J. (2012). Thermal Properties of Epoxy Resin/Filler Hybrid Composites. Polym. Degrad. Stab..

[11] Skeist I. (2011). Handbook of Adhesives.

[12] Luo C., Zuo J., Zhao J. (2013). Synthesis and Property of Epoxy Prepolymer and Curing Agent with High Refractive Index. High Perform. Polym..

[13] Zhang W., Li X., Li L., Yang R. (2012). Study of the Synergistic Effect of Silicon and Phosphorus on the Blowing–Out Effect of Epoxy Resin Composites. Polym. Degrad. Stab..

[14] Toldy A., Szabó A., Novák C., Madarász J., Tóth A., Marosi G. (2008). Intrinsically Flame Retardant Epoxy Resin-Fire Performance and Background–Part II. Polym. Degrad. Stab..

[15] Gaan S., Mauclaire L., Rupper P., Salimova V., Tran T.T., Heuberger M. (2011). Thermal Degradation of Cellulose Acetate in Presence of Bis–Phosphoramidates. J. Anal. Appl. Pyrolysis.

[16] Lin C.H., Chang S.L., Wei T.P., Ding S.H., Su W.C. (2010). Facile, One–Pot Synthesis of Phosphinate–Substituted Bisphenol A and its Alkaline-Stable Diglycidyl Ether Derivative. Polym. Degrad. Stab..

[17] Cakić S.M., Ristić I.S., Jašo V.M., Radičević R.Ž., Ilić O.Z., Simendić J.K.B. (2012). Investigation of the Curing Kinetics of Alkyd–Melamine–Epoxy Resin System. Prog. Org. Coat..

[18] Zheng Y., Chonung K., Jin X., Wei P., Jiang P. (2008). Study on the Curing Reaction, Dielectric and Thermal Performances of Epoxy Impregnating Resin With Reactive Silicon Compounds as New Diluents. J. Appl. Polym. Sci..

[19] Tseng C.H., Hsueh H.B., Chen C.Y. (2007). Effect of Reactive Layered Double Hydroxides on the Thermal and Mechanical Properties of LDHs/Epoxy Nanocomposites. Compos. Sci. Technol..

[20] Shi Y.Q., Fu T., Xu Y.J., Li D.F., Wang X.L., Wang Y.Z. (2018). Novel Phosphorus-Containing Halogen-Free Ionic Liquid Toward Fire Safety Epoxy Resin with Well-Balanced Comprehensive Performance. Chem. Eng. J..

[21] Kou Y.J., Zhou W.Y., Li B., Dong L., Duan Y.E., Hou Q.W., Liu X.R., Cai H.W., Chen Q.G., Dang Z.M. (2018). Enhanced Mechanical and Dielectric Properties of an Epoxy Resin Modified With Hydroxyl-Terminated Polybutadiene. Compos. Part A Appl. Sci..

[22] Laurien M., Demir B., Buttemeyer H., Herrmann A.S., Walsh T.R., Ciacchi L.C. (2018). Atomistic Modeling of the Formation of a Thermoset/Thermoplastic Interphase During Co-curing. Macromolecules.

[23] Zhao M., Meng L.H., Ma L.C., Ma L.N., Yang X.B., Huang Y.D., Ryu J.E., Shankar A., Li T.X., Yan C. (2018). Layer–by–Layer Grafting CNTs Onto Carbon Fibers Surface for Enhancing the Interfacial Properties of Epoxy Resin Composites. Compos. Sci. Technol..

[24] Salmeia K.A., Gaan S. (2015). An Overview of Some Recent Advances in DOPO–derivatives: Chemistry and Flame Retardant Applications. Polym. Degrad. Stab..

[25] Roy S., Maiti S. (1998). Synthesis and Characterization of Thermally Stable Cardo Polyphosphonates. Polymer.

[26] Varganici C.D., Rosu L., Bifulco A., Rosu D., Mustata F., Gaan S. (2022). Recent Advances in Flame Retardant Epoxy Systems From Reactive DOPO-Based Phosphorus Additives. Polym. Degrad. Stab..

[27] Bifulco A., Parida D., Salmeia K.A., Nazir R., Lehner S., Stämpfli R., Markus H., Malucelli G., Branda F., Gaan S. (2020). Fire and Mechanical Properties of DGEBA-Based Epoxy Resin Cured With a Cycloaliphatic Hardener: Combined Action of Silica, Melamine and DOPO-Derivative. Mater. Des..

[28] Carja I.D., Serbezeanu D., Vlad-Bubulac T., Hamciuc C., Coroaba A., Lisa G., López C.G., Soriano M.F., Pérez V.F., Sánchez M.D.R. (2014). A Straightforward, Eco-Friendly and Cost-Effective Approach Towards Flame Retardant Epoxy Resins. J. Mater. Chem. A.

[29] Chen L., Wang Y.Z. (2010). Aryl Polyphosphonates: Useful Halogen-Free Flame Retardants for Polymers. Materials.

[30] Varganici C.D., Rosu L., Lehner S., Hamciuc C., Jovic M., Rosu D., Mustata F., Gaan S. (2021). Semi–Interpenetrating Networks Based on Epoxy Resin and Oligophosphonate: Comparative Effect of Three Hardeners on the Thermal and Fire Properties. Mater. Des..

[31] Reinprecht L., Hulla M. (2015). Colour Changes in Beech Wood Modified With Essential Oils Due to Fungal and Ageing-Fungal Attacks with Coniophora Puteana. Drewno.

[32] Cogulet A., Blanchet P., Landry V. (2016). Wood Degradation Under UV Irradiation: A Lignin Characterization. J. Photochem. Photobiol. B Biol..

[33] Gu X., Stanley D., Byrd W.E., Dickens B., Vaca-Trigo I., Meeker W.Q., Nguyen T., Chin J.W., Martin J.W. (2009). Linking Accelerated Laboratory Test with Outdoor Performance Results for a Model Epoxy Coating System. Service Life Prediction of Polymeric Materials—Global Perspectives.

[34] Rosu D., Rosu L., Cascaval C.N. (2008). Effect of Ultraviolet Radiation on Vinyl Ester Network Based on Bisphenol A. J. Photochem. Photobiol. A Chem..

[35] Larché J.F., Bussière P.O., Thérias S., Gardette J.L. (2012). Photooxidation of Polymers: Relating Material Properties to Chemical Changes. Polym. Degrad. Stab..

[36] Silverstein R.M., Webster F.S., Kiemle D.J. (2005). Spectrometric Identification of Organic Compounds.

[37] Field L.D., Li H.L., Magill A.M. (2020). Organic Structures from Spectra.

[38] Delor-Jestin F., Drouin D., Cheval P.Y., Lacoste J. (2006). Thermal and Photochemical Ageing of Epoxy Resin—Influence of Curing Agents. Polym. Degrad. Stab..

[39] Vera-Graziano R., Hernandez-Sanchez F., Cauich-Rodriguez J.V. (1995). Study of Crosslinking Density in Polydimethylsiloxane Networks by DSC. J. Appl. Polym. Sci..

