# Supplementary material for: Effect of Hardener Type on the Photochemical and Antifungal Performance of Epoxy and Oligophosphonate S–IPNs
Source: Polymers (Basel). 2022 Sep 9;14(18):3784. doi: 10.3390/polym14183784 (PMC9506056; doi:10.3390/polym14183784)
Supplement: Supplementary file 1 [file polymers-14-03784-s001.zip › polymers-1895639-supplementary.pdf]

Supplementary Materials

Effect of Hardener Type on the Photochemical and Antifungal Performance of Epoxy and Oligophosphonate S–IPNs

Cristian-Dragos Varganici, Liliana Rosu *, Dan Rosu *, Corneliu Hamciuc, Irina Rosca and Ana-Lavinia Vasiliu

"Petru Poni" Institute of Macromolecular Chemistry, 41A Grigore Ghica Voda Alley, 700487 Iasi, Romania

* Correspondence: lrosu@icmpp.ro (L.R.); drosu@icmpp.ro (D.R.)

1. Total number of pages: S1 – S6 (6 pages)
2. Total number of Figures: 2
3. Total number of Tables: 4
4. Total number of References: 1

S1. EXPERIMENTAL SECTION

S1.1. Synthesis of Intermediates 1 and 2 (the Precursor to the OP).

The syntheses of intermediate 1 and the precursor to the OP (intermediate 2) were reported in a recent article.[1] According to the procedure 4,4′–terephthalylidene–bis(p–hydroxyaniline) (intermediate 1) was synthesized through the reaction of 4–aminophenol (0.2 mol), terephthalaldehyde (0.1 mol). A catalytic amount of 4–toluenesulfonic acid was added together with the mixture into 300 mL dry ethanol (Scheme S1), followed by 3 h reflux under stirring and cooling at room temperature. Afterwards, the resulting compound, first separated by precipitation over a water–ice mixture, then filtered, washed with distilled water and vacuum dried, was recrystallized from toluene.

The synthesis of bis((6–oxido–6H–dibenz[c,e][1,2]oxaphosphorinyl) –(4–hydroxyaniline)–methylene)–1,4–phenylene (intermediate 2 or the precursor to the OP), was undertaken by reacting intermediate 1 with DOPO. Intermediate 1 (14.62 g, 0.0462 mol), DOPO (20 g, 0.0926 mol) and dried ethanol (103 mL) were mixed and stirred into a round flask, equipped with magnetic stirrer and condenser, at 50 °C for 12 h under nitrogen gas flow. A 90 % yield of filtered resulting precipitate was ethanol washed and vacuum dried. FT-IR (KBr, cm^{-1}): 3265 (NH), 3060 (C–H aromatic), 1477 (P–Ar), 1218 and 1142 (P=O), 1043 (P–O–C), 914 (P–O-Ar), 753. ^{1}H–NMR (400 MHz,

DMSO–d6, d, ppm): 8.50 (m, 2H), 8.17 (m, 4H), 7.88 (m, 2H), 7.68 (m, 2H), 7.56 (m, 2H), 7.42 (m, 2H), 7.34 (m, 4H), 7.18 (m, 2H), 6.54 (m, 8H), 6.1 and 5.6 (m, 2H, N–H), 5.4 and 4.9 (m, 2H, CH–P).

S1.2. Synthesis of the OP.

In order to synthesize the OP, equimolecular quantities of the previously discussed intermediate 2 and phenylphosphonic dichloride were subjected to solution polycondensation (Scheme S1). In a flask having a reflux condenser, magnetic stirrer and nitrogen inlet and outlet there were mixed intermediate 2 (7.48 g, 0.01 mol) with N–methyl–2–pyrrolidone (NMP) (30 mL) and triethylamine (3 mL) to obtain a homogeneous solution. The phenylphosphonic dichloride (1.95 g, 0.01 mol) was added slowly for 30 minutes under stirring. The reaction vessel, was first heated in an oil bath at 50 °C under vigorous stirring for 8 h, then further cooled to room temperature and poured into methanol, filtered and re–dissolved in NMP. The OP, a solid powder (yield 94%), was obtained by precipitation in water, washed with water and vacuum oven dried at 60 °C for 24 h. The structural characterization of the OP, EP and S–IPNs, is detailed in a recent article.[1]

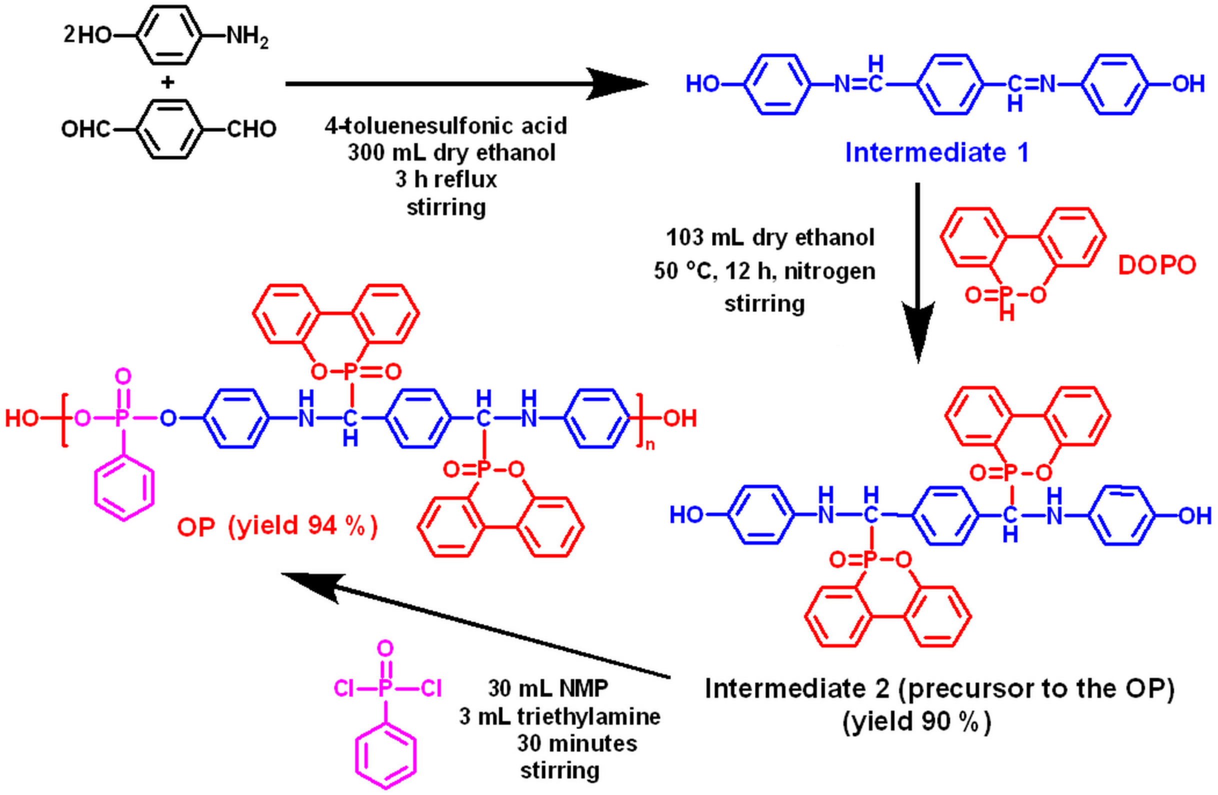

Scheme S1. Synthesis of the intermediates to the OP and the OP

S2. RESULTS AND DISCUSSION

Table S1. **The Differences $\Delta E_{L^*a^*b^*}$ and $\Delta E_{L^*C^*h^*}$ Calculated at Different Exposure Times for the Studied S–IPNs.**

SIPN	Irradiation time [h]	$\Delta E_{L^*a^*b^*}$	$\Delta E_{L^*C^*h^*}$
EP–OP–DDS	100	20.14	19.79
	200	24.0	25.70
	300	27.52	25.63
	400	31.34	29.75
	500	37.03	35.64
EP–OP–CYDM	100	21.90	*25.77*
	200	22.68	18.34
	300	26.89	26.28
	400	26.32	26.32
	500	28.03	32.85
EP–OP–8CH_2DA	100	29.12	27.94
	200	32.80	32.44
	300	32.0	30.82
	400	34.42	33.01
	500	36.58	35.56

Table S2. Color Modifications During UV Irradiation

S–IPN	Non irradiated	Irradiated 500 h	
EP–OP–DDS	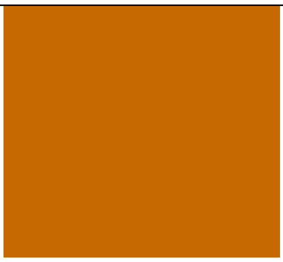<br>L^*=54.3, a^*=30.7, b^* = 79.8,<br>C^* =85.4, h^* = 69	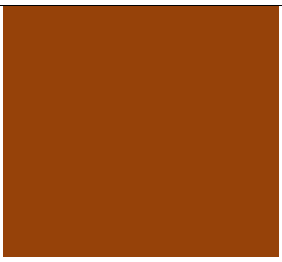<br>L^* = 38.7, a^* = 32.6, b^* =46.3<br>C^* = 56.7, h^* = 54.9	$\Delta E_{L^*a^*b^*}$= 37.0<br>$\Delta E_{L^*C^*h^*}$= 35.6
EP–OP–CYDM	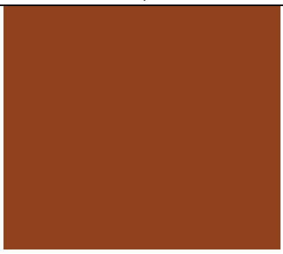<br>L^* =33.8, a^*=31.3, b^* = 35.5,<br>C^* = 47.3, h^* =48.5	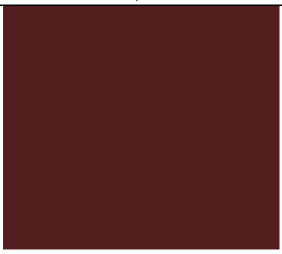<br>L^* = 19.8, a^* = 24.4, b^* =<br>12.2,<br>C^* = 27.3, h^* = 26.5	$\Delta E_{L^*a^*b^*}$= 28.0<br>$\Delta E_{L^*C^*h^*}$= 32.8
EP–OP–8CH$_2$DA	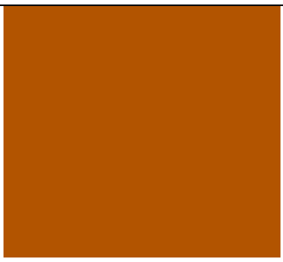<br>L^* =.46.7, a^* = 34.3, b^* =64.7,<br>C^* = 73.3, h^* = 62.1	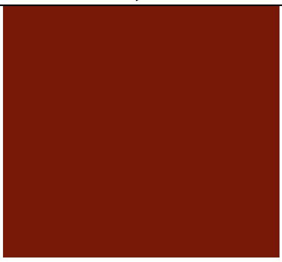<br>L^* =25.8, a^* = 40.2, b^* =35.2,<br>C^* = 53.4, h^* = 41.2	$\Delta E_{L^*a^*b^*}$= 36.6<br>$\Delta E_{L^*C^*h^*}$= 35.6

S2.1. Mass Variation During UV Irradiation.

Mass variations of the samples were also measured during irradiation. The results are summarized in Figure S1. From Figure S1 it can be seen that the highest mass losses were registered in the first 200 h of UV exposure. The mass loss ranged between 6.6% for EP–OP–DDS and 14.2% for EP–OP–8CH$_2$DA, EP–OP–CYDM having a similar mass loss (6.7%) as EP–OP–DDS. After 200 h of exposure there is a mass stabilizing tendency, at the end of the 500 h of UV exposure the values being: 6.8% for EP–OP–DDS and EP–OP–CYDM and 14.3% for EP–OP–8CH$_2$DA, respectively.

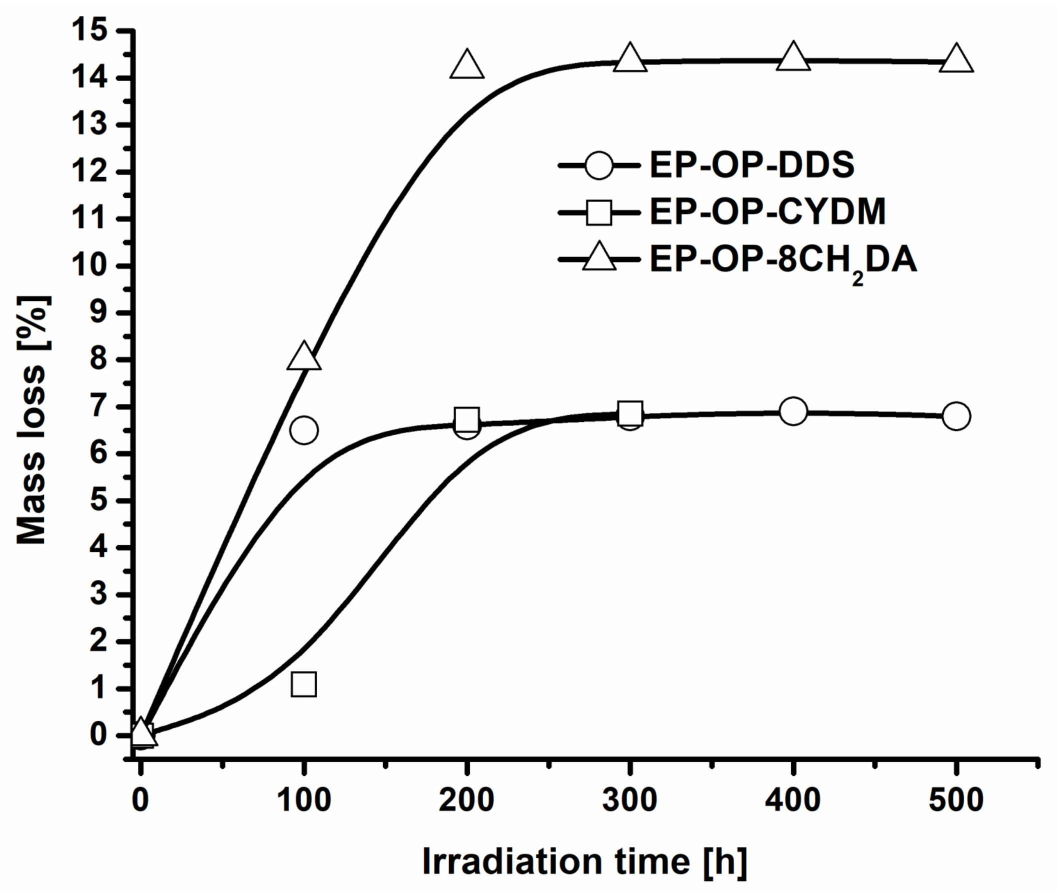

Figure S1. Variation of mass loss with irradiation time.

S.2.2. Thermal Degradation Sudy.

The thermal stability of the initial S–IPNs was investigated by thermogravimetric analysis (TGA), assessed via the temperature corresponding to 5% mass loss ($T_{5\%}$) in nitrogen (N_2) and air atmospheres (50 mL min^{-1}) at a heating rate of 10 °C min^{-1}, up to 700 °C, and reported in a recent article.[1] A summary of the thermal properties extracted from TGA data is depicted in Table S3. The S–IPNs thermal stabilities were dictated by the chemical nature of the curing agent (EP–OP–DDS > EP–OP–CYDM > EP–OP–8CH_2DA). The evolved gases analyses were undertaken with the aid of coupled TG–FT-IR and Py–GC–MS techniques and a thermal degradation mechanism was proposed. The compactness of the S–IPNs lead to a decrease in the number and rate of volatiles evolvement compared to the pristine OP.

Table S3. Thermogravimetric analysis (TGA) Parameters of the S–IPNs.

Sample	Atmosphere	$T_{5\%}$ (°C)	Thermal degradation stages				T_{endset} (°C)	R (%)
			T_{max1} (°C)	T_{max2} (°C)	T_{max3} (°C)	T_{max4} (°C)		
EP–OP–DDS	Air	315	360	550	–	–	575	13.31
	N_2	321	363	387	–	–	410	34
EP–OP–CYDM	Air	213	335	552	–	–	580	7.13
	N_2	262	344	387	458	–	473	12.26
EP–OP–8CH_2DA	Air	225	340	435	555	–	587	3.07
	N_2	249	347	462	–	–	485	13.04

$T_{5\%}$ – temperature corresponding to 5% mass loss;

Table S4. Color Parameters Values Modification after Fungal Attack.

	C.cladosporioides				*P.chrysogenum*				*A.brasiliensis*			
	L*	a*	b*	E*	L*	a*	b*	E*	L*	a*	b*	E*
EP-OP-DDS	0.26	65.8	-23.8	70.0	-5.9	61.2	-50.1	61.5	34.8	64.8	-34.5	73.6
EP-OP-CYDM	-10.3	-10.6	-27.3	31.1	-8.6	-21.2	-27.9	22.9	-10.9	-7.5	-25.1	13.2
EP–OP–8CH_2DA	-31	-10.3	-53.1	62.3	40.3	-29.3	76.4	49.8	-29.5	-1.7	-48.3	29.5

T_{max} – temperature corresponding to the highest mass loss rate per each thermal degradation stage;

T_{endset} – temperature corresponding to the endset of thermal degradation;

R – residue mass remained at 700 °C;

REFERENCES

(1) Varganici, C. D.; Rosu, L.; Lehner, S.; Hamciuc, C. ; Jovic, M. ; Rosu, D. ; Mustata, F. ; Gaan, S. Semi–Interpenetrating Networks Based on Epoxy Resin and Oligophosphonate: Comparative Effect of Three Hardeners on the Thermal and Fire Properties. *Mater. Design* **2021**, *212*, 110237. DOI: 10.1016/j.matdes.2021.110237
